# Hard to Find, Hard to Ride: Findings From Developing a Statewide Transportation Database

**DOI:** 10.1093/geroni/igaf122.3782

**Published:** 2025-12-31

**Authors:** Elizabeth Pinyan, Shannen Johnson, Kristel Robison, Daniel Levitt, Jonathon Weisenfeld

**Affiliations:** UNC-Chapel Hill, Chapel Hill, North Carolina, United States; UNC-Chapel Hill, Chapel Hill, North Carolina, United States; UNC-Chapel Hill, Chapel Hill, North Carolina, United States; UNC-Chapel Hill, Chapel Hill, North Carolina, United States; UNC-Chapel Hill, Chapel Hill, North Carolina, United States

## Abstract

Transportation access is critical for health, independence, and social connection, especially for older adults. Yet finding alternatives to driving is often difficult, even when services exist. In North Carolina, being unaware of available services is the top barrier to access for older adults. To address this gap, we developed a searchable database of non-fixed-route transportation services across all 100 North Carolina counties. We identified providers (n = 116) and services (n = 298) and gathered details on service area, eligibility, trip purpose, accessibility, transportation type, and scheduling. Reviewing provider websites revealed significant barriers to finding and understanding transportation information. Accurate details were rarely available in one place and often required piecing together multiple sources and contacting providers directly. Common issues included vague eligibility criteria (e.g., “elderly” instead of a specific age), emphasis on administrative details over practical rider information, and frequent use of transportation jargon without explaining what it means for the rider. Transportation services go unused when people do not know they exist, how to use them, or do not see themselves as users. When websites are not user-friendly, potential riders may assume the service itself will be difficult to use. In North Carolina, older adults most often rely on family, friends, or websites for information, highlighting the value of accessible transportation information for older adults and their support systems. This database is an important step forward, but broader improvements are needed. Future directions include formal analysis of provider websites and development of best-practice guidelines for age- and accessibility-friendly transportation communication.

